# Comparison of dose calculations between pencil-beam and Monte Carlo algorithms of the iPlan RT in arc therapy using a homogenous phantom with 3DVH software

**DOI:** 10.1186/1748-717X-8-284

**Published:** 2013-12-05

**Authors:** Jin Ho Song, Hun-Joo Shin, Chul Seung Kay, Soo-Min Chae, Seok Hyun Son

**Affiliations:** 1 Department of Radiation Oncology, Seoul St. Mary’s hospital, College of Medicine, The Catholic University of Korea, Seoul, Korea; 2 Department of Radiation Oncology, Incheon St. Mary’s hospital, College of Medicine, The Catholic University of Korea, Incheon, Korea; 3 Department of Radiation Oncology, Cheju Halla General Hospital, Jeju, Korea

**Keywords:** Arc therapy, 3DVH, Monte Carlo, Pencil-beam

## Abstract

**Background:**

To create an arc therapy plan, certain current general calculation algorithms such as pencil-beam calculation (PBC) are based on discretizing the continuous arc into multiple fields to simulate an arc. The iPlan RT™ treatment planning system incorporates not only a PBC algorithm, but also a more recent Monte Carlo calculation (MCC) algorithm that does not need beam discretization. The objective of this study is to evaluate the dose differences in a homogenous phantom between PBC and MCC by using a three-dimensional (3D) diode array detector (ArcCHECK™) and *3DVH* software.

**Methods:**

A cylindrically shaped ‘target’ region of interest (ROI) and a ‘periphery ROI’ surrounding the target were designed. An arc therapy plan was created to deliver 600 cGy to the target within a 350° rotation angle, calculated using the PBC and MCC algorithms. The radiation doses were measured by the ArcCHECK, and reproduced by the *3DVH* software. Through this process, we could compare the accuracy of both algorithms with regard to the 3D gamma passing rate (for the entire area and for each ROI).

**Results:**

Comparing the PBC and MCC planned dose distributions directly, the 3D gamma passing rates for the entire area were 97.7% with the gamma 3%/3 mm criterion. Comparing the planned dose to the measured dose, the 3D gamma passing rates were 98.8% under the PBC algorithm and 100% under the MCC algorithm. The difference was statistically significant (*p = 0.034*). Furthermore the gamma passing rate decreases 7.5% in the PBC when using the 2%/2 mm criterion compared to only a 0.4% decrease under the MCC. Each ROI as well as the entire area showed statistically significant higher gamma passing rates under the MCC algorithm. The failure points that did not satisfy the gamma criteria showed a regular pattern repeated every 10°.

**Conclusions:**

MCC showed better accuracy than the PBC of the iPlan RT in calculating the dose distribution in arc therapy, which was validated with the ArcCHECK and the *3DVH* software. This may suggest that the arc step of 10° is too large in the PBC algorithm in the iPlan RT.

## Background

Rotational arc radiation therapy has attracted great interest in the modern radiation therapy era. Several techniques such as dynamic arc therapy, intensity-modulated arc therapy (IMAT), volumetric arc therapy (VMAT) and helical tomotherapy have been developed [1-3]. These techniques are currently used widely, especially in prostate, head and neck, and brain tumors [4]. As the technique of rotational arc therapy advances, more precise and conformal radiation dose delivery has become possible. However regardless of how the radiation therapy machine delivers conformal doses, the accuracy of the planning algorithm and quality assurance (QA) should be an essential prerequisite.

Classically, to calculate the dose distribution of an arc therapy plan, most treatment planning systems (TPS) discretize the continuous arc, thereby effectively using several beams to simulate an arc. For example, to calculate a 180° arc, the pencil-beam calculation (PBC) algorithm of iPlan RT™ (BrainLAB, Heimstetten, Germany) planning system uses 19 beams, which are each separated from neighboring beams by a beam angle of 10°. This method is not continuous, and it is sometimes necessary to increase the number of beams to improve the accuracy of the dose distribution [5]. On the other hand, the accuracy of the Monte Carlo dose calculation (MCC) method depends on the number of simulated histories rather than the number of beams. Therefore, the MCC method will be more accurate than current TPSs for arc therapy [6].

In spite of the many reports that have been published to show the superior accuracy of the MCC method with respect to that of other methods [5-10], several studies comparing the accuracy of the PBC and MCC methods for arc therapy have not shown the possible errors that can result from discretizing the continuous arc, and have only focused on the errors occurring in the inhomogeneous condition. Using both homogeneous and inhomogeneous phantoms, Chow et al. compared the PBC, collapsed cone convolution (CCC), and MCC (EGS4-based DOSXYZ code) of the Theraplan Plus™ (MDS Nordian, Ottawa, Canada) TPS when they are applied in arc therapy [5]. For homogenous phantoms, the PBC-measured and CCC-measured doses agreed well (~2% dose error) with predictions of the MCC and the TPS planned dose. However, for the dose distribution in the inhomogeneous phantom, only the MCC agreed well with measurements within a 2% error, while the PBC and CCC either underestimated or overestimated the dose. Petoukhova et al. also compared the PBC with the MCC of iPlan RT both in homogenous and inhomogeneous phantoms for conformal radiotherapy, IMRT, and dynamic arc therapy [6]. They also concluded that the PBC and MCC results agreed well with the measured dose in homogenous phantoms, but not with that in inhomogeneous phantoms. The commonality of these studies is that they only measured the delivered dose with 1) an ion-chamber or 2) a film and a 2D diode array detector.

There are plenty of QA methods for evaluating the dosimetric accuracy of arc therapy such as gel dosimetry, film dosimetry and diode array detectors [11,12]. The diode array detector has been used by a number of institutions because it can be performed relatively quickly and easily, and because it is able to measure each beam. Recently, novel three-dimensional (3D) diode array detectors have emerged to overcome the limitation of existing two-dimensional (2D) detectors which can miss a large fraction of the lateral beams because of their individual planar design [1,12]. This disadvantage of 2D detectors can be maximized in arc therapy. Delta4 (Scandidos, Uppsala, Sweden) and ArcCHECK (SunNuclear, Melbourne, FL, USA) are representative examples of 3D detectors and are commonly used in IMRT and arc therapy QA [1,12-14].

In spite of the advancement of delivery QA devices, describing and interpreting the QA result is still difficult. The most commonly used concept for determining whether the measured result is appropriate is gamma analysis, which uses two concepts: dose difference and distance-to-agreement (DTA) [15]. The rate that satisfies a gamma criterion is called the gamma passing rate. Although, several gamma criteria and acceptance levels have been used, a 3%/3 mm criterion and a 90% acceptance level are commonly recommended in planar dosimetry, as suggested by AAPM Task Group 119 [16]. However, the clinical significance of the criteria for this assessment is not clear. A higher gamma passing rate does not always ensure safe radiation treatment. The magnitude and location of the dose error are also very important.

The commercially available *3DVH* software program (SunNuclear) can overcome some of the disadvantages of the planar gamma analysis concepts. With the aid of this software, the full 3D dose distribution can be reconstructed based on the measured data, and it can be compared to the TPS planning dose. In addition, a dose-volume histogram (DVH) for each target and each region of interests (ROI) can be drawn [17,18].

In this study, we evaluated the accuracy of the MCC and the PBC algorithm of the iPlan RT software in arc therapy by using a 3D volumetric diode array detector and the *3DVH* software. We used a homogenous phantom to focus on the dose differences that will occur by discretizing a continuous arc. We compared not only the 3D gamma passing rate but also several DVH-based parameters of the MCC to those of the PBC.

## Methods

### Treatment design and planning

First we designed a simple radiation treatment plan to evaluate not only the target dose but also the peripheral dose. Homogenous CT images of a virtual ArcCHECK (provided by SunNuclear) with the same characteristics (cylindrical shape, diameter: 26.5 cm, length: 27.0 cm) as those of ArcCHECK were used. This CT set included 165 images at a slice thickness of 1.25 mm.

**Figure 1:**
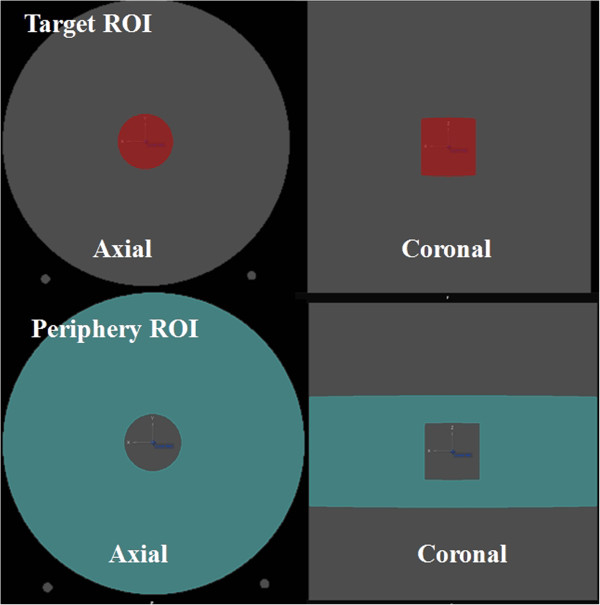
Axial and coronal images of the ‘target ROI and the ‘periphery ROI.

Two ROIs were designed. A circle with a 5 cm diameter was contoured with an isocenter as the center of the axial CT image, and it was named the “target ROI”. The height of the target ROI was also 5 cm, so the target had a cylindrical shape. Another ROI was designed, which was named the “periphery ROI”. It is a donut-shaped ROI on the axial CT image with a 21.5 cm thickness, which is made by taking a circle with a 26.5 cm diameter and then excluding the target ROI. The height of the periphery ROI was 10 cm, so it was a circle of 26.5 cm diameter on the axial CT image in which the target ROI does not exist. The axial and coronal images of these ROIs are shown in Figure 1. The volume of the target and periphery ROIs were 106.9 cc and 5432.1 cc, respectively.

For this study, we used iPlan RT™ (version 4.1.2, BrainLAB). We prescribed 600 cGy to 95% of the target ROI. The multi-leaf collimator (MLC) margins were set to 3 mm from the target in the beams-eye-view. A dynamic conformal arc with a rotation angle of 350° (from 185° to 175°) in the clockwise direction was used as the treatment mode.

The plan was calculated using both a PBC algorithm and a MCC algorithm. The iPlan RT itself provides both a PBC and MCC algorithm. The PBCs were performed with an arc step of 10°. After the plan using the PBC algorithm had been completed, this plan was recalculated by applying the MCC algorithm. The commercial MCC algorithm in iPlan RT is based on XVMC (X-ray voxel Monte Carlo) code developed by Kawrakow et al. and Fippel [19,20]. The MCC algorithm in iPlan RT has been validated by Petoukhova et al. and Fragoso et al. [6,10]. The dose calculation resolution was 1 mm for both algorithms. The mean variance which estimates the statistical uncertainty of the MCC was set to 2%. The dose result type and the MLC modeling type were chosen as ‘dose to medium’ and ‘accuracy optimized’. Whether the plan was calculated by PBC and MCC algorithm, the radiation that is actually delivered is same.

After the completion of both PBC and MCC calculation, we compared the dose distributions between both algorithms, with the aid of *3DVH* software. The global gamma passing rates were analyzed with 3%/3 mm and 2%/2 mm gamma criteria and a threshold of 5% of the maximal dose. We used the gamma passing rates for the plan comparison because the *3DVH* software does not support use of a simple dose difference criterion (such as a 3%/0 mm criterion), and also to allow the dose errors to be more easily compared by applying the same criteria.

### Dose delivery and verification with ArcCHECK

All tests were carried out with a Novalis™ (BrainLAB, Heimstetten, Germany, and Varian Medical Systems, Palo Alto, CA) accelerator with a 6 MV X-ray energy and a micro-MLC.

The delivered dose was measured with ArcCHECK which is a cylindrical acrylic 3D diode array detector. It contains 1386 diodes in a helical arrangement at intervals of 10 mm and with a diameter of 21 cm. The physical depth of each diode is 2.9 cm. The ArcCHECK was calibrated with an absolute dose of 200 cGy with 10 × 10 cm^2^ field size at gantry angle 0° before this study. The ArcCHECK has a central cavity into which various detectors can be plugged. An EXRADIN A16-micro ion-chamber (Standard Imaging, Middleton, WI, USA) was inserted in an acryl insert (CavityPlug™) that can directly measure the isocenter dose. The isocenter dose was measured with a SUPERMAX electrometer (Standard Imaging). During this whole study, the ArcCHECK was operated with the plug and the ion-chamber inside. All measurements were repeated three times sequentially.

First, we analyzed the 2D gamma passing rates at the level of diodes. For this analysis, a SNC Patient™ (v.6.1.1, SunNuclear) software program was used. A digital imaging and communications in medicine (DICOM) dose file of the treatment plan is imported in this software and the dose corresponding to diode detector locations is extracted for comparing the calculated dose to measured dose. With this software, we obtained the gamma passing rate of each plan at the level of diodes. We analyzed the ‘global’ gamma passing rate in which the percentage dose difference is calculated relative to the maximum dose in the measurement plane. Two gamma criteria, 3%/3 mm and 2%/2 mm, were used with a threshold of 5% of the maximal dose.

### Analysis of 3D gamma passing rate with 3DVH

The doses measured using the ArcCHECK diodes were imported into the *3DVH* software along with four other DICOM files from the TPS: the treatment plan, the CT images, the outlined structures, and the planned dose files. The *3DVH* software can calculate the discrepancies between the planned dose and the measured planar dose distributions, and then translate the discrepancies to the calculated dose of the patient. We will refer to the resulting calculated dose of the patient as the ‘estimated dose’. This process is done using an algorithm called ‘planned dose perturbation’ or PDP™. Before comparing the planned and measured doses, interpolation is needed to calculate the dose between the diodes because of the sparse diode arrangement. This interpolation process is referred to by the developers as ‘Smaterpolation’. The *3DVH* software compares the estimated dose with the planned dose, and calculates the 3D gamma passing rate not only for the whole volume but also for each of the corresponding ROIs. Further details and validation of the *3DVH* software are explained in Refs.18, 21, and 22 [18,21,22].

In our study, the estimated doses by the *3DVH* software are represented by ion chamber correction doses, which are corrected by the ratio between the ion-chamber measured isocenter dose and isocenter dose estimated by *3DVH* based on diode measurements. The plan doses calculated using the PBC and the MCC were compared to the corresponding estimated dose respectively, and the 3D gamma passing rate of each ROI was calculated. For this 3D gamma analysis, the global gamma indices were calculated with two gamma criteria (3%/3 mm and 2%/2 mm) with a threshold of 5% of the global maximal dose.

The gamma passing rate of the PBC and MCC were compared and the difference was analyzed using the Mann–Whitney *U*-test. The statistical analysis was performed using STATA/IC ver.12 software (StataCorp, College Station, TX, USA).

### Comparison of DVH-based parameters

A great advantage of the *3DVH* software is that various DVH-based parameters can be estimated for all ROI structures. Several parameters are calculated based on the measured data, and they can be compared to the same parameters in the plan. During this process, we can see how the discrepancies of the planned and measured data (e.g. the gamma passing rate) influence the clinical parameters (e.g. the maximal dose and mean dose). We obtained six parameters for each ROI and for the entire plan: maximal dose, mean dose, the dose covering 99% of the volume (D99), the dose covering 90% of the volume (D90), the dose covering 50% of the volume (D50) and the dose covering 1% of the volume (D1). For each parameter, the dose deviation (%) expressed as an absolute value, was calculated using the following formula.

$$\begin{array}{l} \text{Dose}\;\text{Deviation}\;\left(\%\right)=\frac{\left|\text{Estimated}\;\text{dose}-\text{Planned}\;\text{dose}\right|}{\text{Planned}\;\text{dose}}\\ \;\times 100\;\left(\%\right) \end{array}$$

## Results

### Reference study with static beams

Before evaluating the rotational arc therapy, we performed a reference study with parallel-opposing static beams. The field size was 10 × 10 cm^2^ with no blocking area, and the gantry angles were 90° and 270°. We prescribed 200 cGy to 95% of the target ROI. This plan was calculated using the PBC algorithm first, and then recalculated using the MCC algorithm of the iPlan RT™ (version 4.1.2). The planned dose distributions were compared using *3DVH* software with the same gamma criteria mentioned above. The gamma passing rates were 100%, 98.7%, and 98.8% for the target ROI, the periphery ROI, and the entire area with the gamma 3%/3 mm criterion. With the 2%/2 mm criterion, the gamma passing rates were 100%, 96.6%, and 96.7%, respectively.

**Figure 2:**
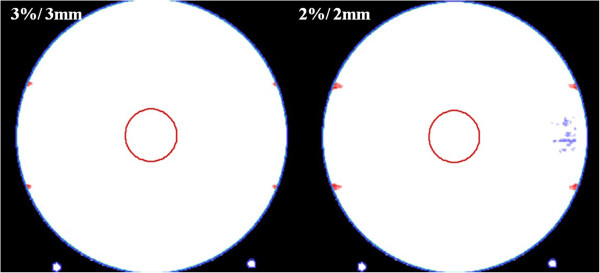
**Comparison between the pencil-beam calculation dose distribution and the Monte Carlo calculation dose distribution of the reference plan with static beams.** The points that failed to satisfy the gamma criteria (Left: 3%/3 mm, Right: 2%/2 mm) are shown.

Reference measurements with ArcCHECK device were also performed using the parallel-opposing static plan, and these results were also analyzed using *3DVH* software and are shown in Table 1. The gamma passing rates of the target ROI were 100% in both the PBC and MCC even with the 2%/2 mm gamma criterion. However, the gamma passing rate of the periphery ROI were 94.2% in the PBC and 97.7% in the MCC with the 2%/2 mm gamma criterion. The difference was statistically significant (*p = 0.046*) according to the Mann–Whitney *U*-test. The points that failed to satisfy the gamma criteria (3%/3 mm and 2%/2 mm) are shown in Figure 2.

**Table 1:** 3D global gamma analysis of the reference plan with static beams

**Gamma 3%/3 mm**	**Gamma passing rate (%)**		***p value***
	**PBC**	**MCC**	
Target ROI	100 ± 0.00	100 ± 0.00	*1.000*
Periphery ROI	98.00 ± 0.10	99.70 ± 0.00	*0.037*
Entire area	98.10 ± 0.10	99.70 ± 0.00	*0.034*
**Gamma 2%/2 mm**	**PBC**	**MCC**	***p value***
Target ROI	100 ± 0.00	100 ± 0.00	*1.000*
Periphery ROI	94.20 ± 0.30	97.77 ± 0.12	*0.046*
Entire area	94.47 ± 0.31	97.90 ± 0.10	*0.050*

*Abbreviations*: *PBC* Pencil-beam calculation, *MCC* Monte Carlo calculation.

Two gamma criteria (3%/3 mm and 2%/2 mm) with a threshold of 5% of maximal dose were used. The 3D gamma passing rate of the entire area and of each region of interest (target and periphery ROI) obtained through the pencil-beam calculation (PBC) are compared with those obtained through the Monte Carlo calculation (MCC) using the Mann–Whitney *U*-test. The dose was measured three times, and is shown as mean ± the standard deviation.

### Comparison between the PBC and MCC dose distributions in the arc plan

**Figure 3:**
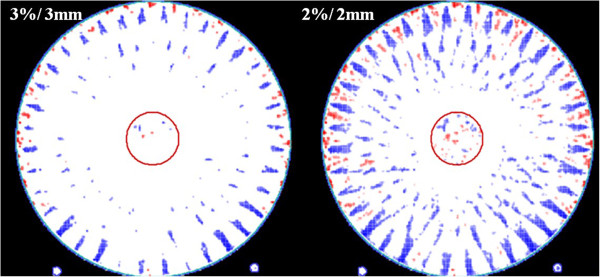
**Comparison between the pencil-beam calculation dose distribution and the Monte Carlo calculation dose distribution of the arc plan.** The points that failed to satisfy the gamma criteria (Left: 3%/3 mm, Right: 2%/2 mm) are shown.

With the aid of *3DVH* software, we directly compared the PBC and MCC-calculated dose distribution. The 3D gamma passing rates were 97.7%, 96.6% and 96.7% for the target ROI, periphery ROI and the entire area with the gamma 3%/3 mm criterion. The gamma passing rates decreased to 86.9%, 89.2% and 89.0%, respectively, when 2%/2 mm criterion was used. The points that failed to satisfy the gamma criteria (3%/3 mm and 2%/2 mm) are shown in Figure 3.

### Analysis of 2D gamma passing rates at the level of diodes with ArcCHECK

The gamma passing rates at the level of diodes are shown in Table 2. For the gamma 3%/3 mm criterion, the gamma passing rates were 88.5% in PBC and 97.9% in MCC. The gamma passing rates decreased to 72.1% in PBC and 91.2% in MCC, when 2%/2 mm criterion was applied. However, no matter which criterion was used, the gamma passing rates at the level of diodes were significantly higher in MCC compared to PBC (*p < 0.05*).

**Table 2:** **2D gamma analysis at the levels of diodes with two gamma criteria (3**%**/3 mm and 2**%**/2 mm) in absolute doses with a threshold of 5**%

**Gamma criteria**	**Gamma passing rate (%)**		***p value***
	**PBC plan**	**MCC plan**	
3%/3 mm	88.50 ± 1.91	97.90 ± 1.21	*0.043*
2%/2 mm	72.07 ± 0.40	91.17 ± 0.12	*0.046*

*Abbreviations*: *PBC* Pencil-beam calculation, *MCC* Monte Carlo calculation.

The statistical significance between the pencil-beam calculation (PBC) and the Monte Carlo calculation (MCC) is calculated with the Mann–Whitney *U*-test. The dose was measured three times, and is shown as mean ± the standard deviation.

### Analysis of 3D gamma passing rates with 3DVH

Table 3 shows the 3D gamma analysis results for each ROI and the entire area, which were obtained by comparing the planned dose to the estimated dose using the *3DVH* software. We compare these results between the PBC and MCC algorithms. The gamma passing rate of the entire area was 98.8% under the PBC algorithm and 100.0% under the MCC algorithm when the 3%/3 mm gamma criterion was applied. Although both gamma passing rates were high, the gamma passing rate of the MCC was statistically higher than that of the PBC (*p = 0.034*). Furthermore the gamma passing rate decreases 7.5% in the PBC when using the 2%/2 mm criterion compared to only a 0.4% decrease under the MCC algorithm. Not only the entire area but also each ROI (target and periphery) showed statistically significant higher gamma passing rates under the MCC algorithm.

**Table 3:** **3D gamma analysis with two gamma criteria (3**%**/3 mm and 2**%**/2 mm) in absolute doses with a threshold of 5**%

**Gamma 3%/3 mm**	**Gamma passing rate (%)**		***p value***
	**PBC**	**MCC**	
Target ROI	97.60 ± 0.36	99.80 ± 0.00	*0.037*
Periphery ROI	98.83 ± 0.06	100.00 ± 0.00	*0.034*
Entire area	98.83 ± 0.06	100.00 ± 0.00	*0.034*
**Gamma 2%/2 mm**	**PBC**	**MCC**	***p value***
Target ROI	67.57 ± 1.50	96.20 ± 0.06	*0.034*
Periphery ROI	91.97 ± 0.12	99.70 ± 0.00	*0.034*
Entire area	91.30 ± 0.17	99.60 ± 0.00	*0.034*

*Abbreviations*: *PBC* Pencil-beam calculation, *MCC* Monte Carlo calculation.

The 3D gamma passing rate of the entire area and of each region of interest (target and periphery ROI) are compared between the pencil-beam calculation (PBC) and the Monte Carlo calculation (MCC) with the Mann–Whitney *U*-test. The dose was measured three times, and is shown as mean ± the standard deviation.

When we compare the accuracy between the target ROI and the periphery ROI, the gamma passing rate of the target ROI was lower than that of the periphery ROI for all cases. The gamma passing rates of the periphery ROI were 98.8% (PBC) and 100.0% (MCC) compared to 97.6% (PBC) and 99.8% (MCC) for the target ROI under the gamma 3%/3 mm criterion. The gamma passing rates of the periphery ROI were 91.9% (PBC) and 99.7% (MCC) compared to 67.6% (PBC) and 96.2% (MCC) for the target ROI under the gamma 2%/2 mm criterion.

**Figure 4:**
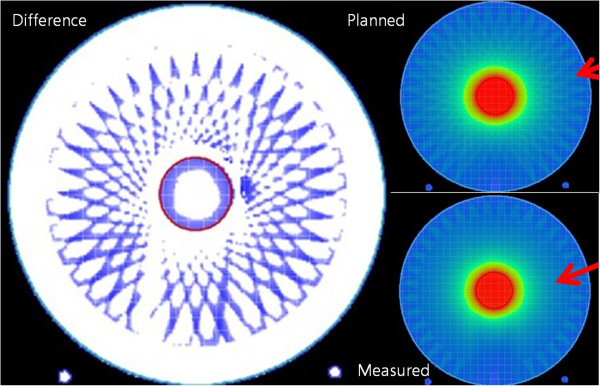
**The points that failed to satisfy the gamma criteria of 2%/2 mm under the pencil-beam calculation algorithm (left).** The dose distribution of the plan calculated by the pencil-beam calculation algorithm is shown in upper right field, and the reconstructed dose distribution (using the *3DVH* software based on the measurements) is shown in the lower right field.

**Figure 5:**
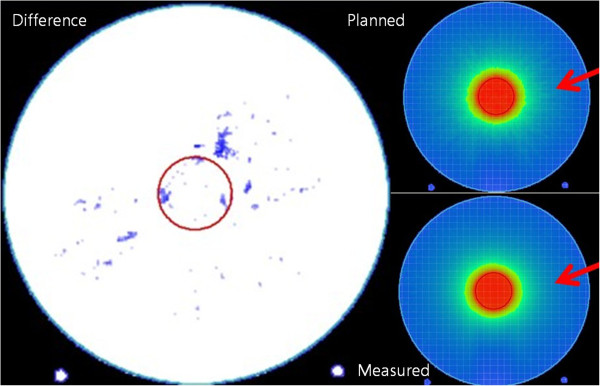
**The points that failed to satisfy the gamma criteria of 2%/2 mm under the Monte Carlo calculation algorithm (left).** The dose distribution of the plan calculated by the Monte Carlo calculation algorithm is shown in upper right field, and the reconstructed dose distribution (using the *3DVH* software based on the measurements) is shown in the lower right field.

Figures 4 and 5 show the points that failed to satisfy the gamma criteria under the PBC and MCC algorithms, respectively. In the PBC algorithm, the failure points are distributed along the rim of the target. For the periphery ROI, the failure points show a regular pattern repeated every 10°. On the other hand, under the MCC algorithm, only a few failure points are scattered in a random fashion in both the target and periphery ROIs.

### Comparison of DVH-based parameters

Table 4 shows the DVH-based parameters in absolute dose, where one is obtained from the planned dose data and the other is estimated by the *3DVH* software based on the ArcCHECK measurements. Table 5 shows the dose deviation in percentage. The mean dose of the target ROI calculated by the PBC algorithm was 627 cGy, similar to that of the 614 cGy estimated by the *3DVH* software. The mean dose of the target ROI calculated by the MCC was 614 cGy, also similar to that of the 622 cGy estimated by the *3DVH* software. The dose deviation was 2% in the PBC and 1% in the MCC with respect to the mean dose. Of the six DVH parameters evaluated in this study, four parameters (mean dose, D99, D90, and D50) showed better accuracy in the MCC than in the PBC in both the target and periphery ROI. On the other hand, for the maximal dose of both ROIs and for the D1 dose of the target ROI, the PBC algorithm showed better agreement.

**Table 4:** **A comparison of six DVH-based dose parameters obtained from the plan and with those estimated by the *3DVH *** **software**

**PBC algorithm**	**Target ROI**		**Periphery ROI**		**Entire area**	
	**Planned dose (cGy)**	**Estimated dose (cGy)**	**Planned dose (cGy)**	**Estimated dose (cGy)**	**Planned dose (cGy)**	**Estimated dose (cGy)**
Max dose	639	630	629	620	639	631
Mean dose	627	614	114	109	124	119
D99	591	571	8	8	8	8
D90	615	595	12	13	12	13
D50	630	619	109	107	111	110
D1	638	629	516	483	630	618
**MCC algorithm**	**Planned dose (cGy)**	**Estimated dose (cGy)**	**Planned dose (cGy)**	**Estimated dose (cGy)**	**Planned dose (cGy)**	**Estimated dose (cGy)**
Max dose	670	646	655	628	670	646
Mean dose	614	622	109	109	119	119
D99	572	588	8	8	8	8
D90	596	603	13	13	13	13
D50	619	627	107	106	110	109
D1	630	640	483	488	618	626

*Abbreviations*: *PBC* Pencil-beam calculation, *MCC* Monte Carlo calculation, *ROI* region of interest, *D99* dose covering 99% of the volume, *D90* dose covering 90% of the volume, *D50* dose covering 50% of the volume, *D1* dose covering 1% of the volume.

The estimated dose is calculated based on the ArcCHECK measured data.

**Table 5:** **A comparison of the dose deviations (%) of six DVH-based dose parameters obtained from the plan and with those estimated by the *3DVH *** **software**

**Dose deviation**	**Target ROI**		**Periphery ROI**		**Entire area**	
	**PBC**	**MCC**	**PBC**	**MCC**	**PBC**	**MCC**
Max dose	1%	4%	1%	4%	1%	4%
Mean dose	2%	1%	4%	0%	4%	0%
D99	3%	1%	0%	0%	0%	0%
D90	3%	1%	8%	0%	8%	0%
D50	2%	1%	2%	1%	1%	1%
D1	1%	2%	6%	1%	2%	1%

*Abbreviations*: *PBC* Pencil-beam calculation, *MCC* Monte Carlo calculation, *ROI* region of interest, *D99* dose covering 99% of the volume, *D90* dose covering 90% of the volume, *D50* dose covering 50% of the volume, *D1* dose covering 1% of the volume.

The estimated dose is calculated based on the ArcCHECK measured data.

In every DVH-based parameter, the dose deviation was below 10% both under the PBC and the MCC algorithm. Dose deviations higher than 5% were only observed in D90 and D1 of the periphery ROI calculated by the PBC algorithm (Table 5).

**Figure 6:**
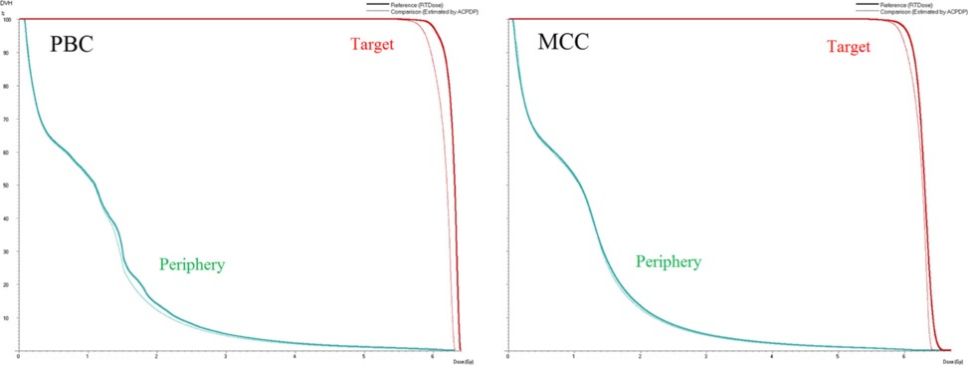
**The dose-volume histograms comparing the plan dose and the *3DVH *estimated dose.** (Left) Pencil-beam calculation (PBC) dose distribution (thick line) vs. *3DVH* estimation (thin line), (Right) Monte Carlo calculation (MCC) dose distribution (thick line) vs. *3DVH* estimation (thin line).

Figure 6 shows the real DVH, comparing the planned dose and the estimated dose. Although no significant differences can be found in several parameters when comparing the PBC and the MCC, the DVH lines showed better agreement in the MCC algorithm compared to the PBC for both ROIs.

## Discussion

Rotational arc therapy has recently been receiving a great deal of attention because of the possibility of acquiring a plan with improved dose distribution, shorter treatment time, and small MU in certain treatment sites. Shepard et al. demonstrated the dosimetric benefits of rotational treatments by summarizing results from an optimization series performed for a C-shaped target [23]. The result showed that each increase in the number of beam angles led to a more homogenous dose in the target and a lower dose to the adjacent structure. The total integral dose was independent of the number of angles. Based on these advantages, rotational arc therapy has been developed to achieve more conformal 3D dose distributions in recent years, such as IMAT, helical tomotherapy and VMAT [1-3]. To use these techniques in clinical practice, the accuracy of the planning algorithm and QA tools are essential.

The usefulness of ArcCHECK, the 3D volumetric diode dosimeter used in this study, has already been validated by other investigators [11,12,14,17,24]. Li et al. performed several tests for the ArcCHECK QA system, and evaluated the suitability of this system for IMRT and VMAT verification [11]. The short term reproducibility, the dose linearity, the dose rate dependence, the dose per pulse dependence, the field size dependence and the out of field dependence were very good, and were satisfied in the clinical QA conditions. Neilson et al. evaluated the accuracy and usefulness of ArcCHECK device with 169 delivery QA plans from 84 patients treated by Hi-ART Tomotherapy [12]. They concluded the ArcCHECK can save resources and provide accurate delivery QA results compared to the QA methods with radiographic film and ion-chamber. Based on these several studies, we incorporated the ArcCHECK QA system for this rotational arc therapy study.

Most commonly used TPSs calculate the dose by evenly dividing the rotating irradiation angles into multiple fields to simulate an arc. The PBC algorithm of the iPlan RT TPS divided the angles into 10° segments [5]. On the other hand, the MCC algorithm basically relies on random sampling and integration [25]. The MCC algorithm of the iPlan RT TPS is based on the XVMC code, which consists of three main components: a virtual energy fluence model [26], a full MC geometry simulation of the photon transport [27], and a patient dose computation [19,20]. Several studies have tested and validated the MCC algorithm of the iPlan RT TPS [6,10].

The accuracy of the MCC compared to the PBC has been reported in many studies [5-10]. However, the comparisons of the MCC and PBC in arc therapy has been limited, and have usually only focused on the discrepancy under inhomogeneous conditions [5,6]. Our result demonstrates that the MCC showed better accuracy than the PBC in an arc plan even under homogenous conditions. When we directly compared the dose distributions of the PBC with those of the MCC, the degree of agreement was only 89% for the entire area as determined by 2%/2 mm gamma analysis. This is despite the fact that the radiation delivery is actually same in both algorithms. This result demonstrates the discrepancy between the two algorithms.

The result of the 2D gamma analysis with ArcCHECK showed that the MCC dose distribution was in better agreement with the measurement than the PBC. The gamma passing rate of the MCC was 19.1% higher than that of the PBC at the levels of ArcCHECK diodes with a 2%/2 mm gamma criterion. With the aid of the *3DVH* software, we were also able to determine that the 3D gamma passing rate was statistically higher in the MCC compared with that in the PBC not only for the entire area, but also for each ROI. The gamma passing rate was 99.8-100% for the MCC compared to 97.6-98.8% for the PBC for a 3%/3 mm gamma analysis. The difference was more prominent when a more stringent gamma criterion (2%/2 mm) was used. Using 2%/2 mm gamma analysis, the gamma passing rate was 96.2-99.7% for the MCC compared with 67.6-91.9% for the PBC. With these results, we can draw the conclusion that the MCC shows better accuracy than the PBC in an arc plan even under homogenous conditions.

The points that failed the gamma criteria in the PBC showed a regular pattern. In the target, the points are distributed at the rim. In the periphery ROI, the points are scattered and repeated at 10° intervals. However, only a few points are scattered in an irregular pattern in the MCC. The patterns observed in our study suggest that the accuracy of the PBC algorithm of iPlan RT is inferior compared with that of the MCC algorithm because of the discretization of the continuous arc, which results in the effective usage of several beams to simulate an arc. To increase the dose distribution accuracy of the PBC, it seems necessary to increase the number of discrete beams for the dose calculation [5]. However, increasing the number of beams will lead to a longer calculation time; for MCC, there is no increase in the calculation time, which can be an inherent advantage of the MCC for arc therapy.

Some precautions must be taken when interpreting our results. First, the magnitude of the discrepancy was not entirely due to discretizing the arc. The reference study with static beams also showed some dose differences between the PBC and MCC algorithms. The degree of agreement between the two dose distributions was determined to be 96.7% for the entire area by means of a 2%/2 mm gamma analysis, for which the errors were observed only in the periphery ROI, and more specifically in the field margin (Figure 2). Based on the ArcCHECK measurement result of the reference study with static beams, we were able to determine that the field margin errors were also from the PBC algorithm because the measurement agreed better with the MCC dose distribution. Although statistically significant differences existed even in the reference study with static beams, the magnitude of the dose difference between the PBC and MCC was much larger in the arc therapy plan (11.0% for the entire area as determined through a 2%/2 mm gamma analysis) compared to the reference plan (3.3% for the entire area as determined through a 2%/2 mm gamma analysis).

Second, this result was based only on the iPlan RT TPS. Therefore, the result of our study should not be generalized to other arc therapy TPSs. For example, Tudor et al. reported that with 51 calculation angles, a dose difference greater than 1% was absent in the Tomotherapy plans studied when the number of calculation angles was increased by a factor of 5 or more [28].

In a study of Petoukhova et al., the authors measured several clinical HybridArc plans (iPlan RT TPS) with an ArcCHECK diode array detector and found that the PBC significantly differed from the ArcCHECK measurements [9]. The gamma passing rate with a 2%/2 mm criterion was 61.5-77.8% in the PBC compared to 91.8-97.5% in the MCC. They also demonstrated points with more than a 2% dose difference. These points were distributed not only in the low-dose region but also within the target, as in our study. However, they could not find a regular pattern among these points, which may be due to the different treatment technique that they used.

In our study, we used the *3DVH* software to obtain the 3D gamma passing rate results not only for the entire area but also for each ROIs. Another great advantage of the *3DVH* software is that several DVH-based parameters can be estimated based on the measured data, and can be compared to that of the plan data. The details of the algorithm and validation of its accuracy have been reported in several reports [12,18,21,22]. Through this process we can obtain information about how the gamma analysis is incorporated into clinical parameters. In our study, it was difficult to find any correlation between the gamma passing rates and the several DVH-based parameters. Although the target ROI gamma passing rate decreases to 67.6% under the PBC algorithm with gamma 2%/2 mm, the dose deviations of all the DVH parameters of the target ROI were just 1-3%. On the other hand, two parameters (D90 and D1) showed more than a 5% dose deviation in the periphery ROI in spite of the 92.0% gamma passing rate. Although it was difficult to draw a conclusion from our study of the correlation between the gamma analysis and several DVH-based parameters, that was also not the aim of this study, and the lack of correlation of the gamma analysis with any of several DVH-based parameters has been demonstrated in several other reports [17,18,21,29]

Through this study, we have also learned that delivery QA using ArcCHECK and *3DVH* software can provide physicians with a great deal of information through a simple and easy procedure, although it remains difficult to suggest specific guidelines, and this question is beyond the scope of this paper.

What is clear in our study is that the MCC showed better accuracy than the PBC of iPlan RT TPS in calculating the dose distribution in arc therapy, which was validated with the ArcCHECK measurement and the *3DVH* software. This may suggest that the arc step of 10° is too large in the PBC algorithm in the iPlan RT TPS.

## Competing interests

The authors declare that they have no competing interests.

## Authors’ contributions

SHS, CSK, and SMC performed the experiment design. SHS and HJS performed data collection. HJS performed the treatment planning and conducted all planning evaluation. JHS and SHS interpreted the data. SHS performed the statistical analysis. JHS drafted the manuscript. All authors read and approved the final manuscript.
